# Activity of rezafungin against *Candida auris*

**DOI:** 10.1093/jac/dkaf124

**Published:** 2025-04-30

**Authors:** Jeffrey B Locke, David Andes, Shawn Flanagan, Mark Redell, Voon Ong, Jalal A Aram, Peter G Pappas, Mariana Castanheira, George R Thompson

**Affiliations:** Cidara Therapeutics, Inc., San Diego, CA, USA; University of Wisconsin-Madison, Madison, WI, USA; Cidara Therapeutics, Inc., San Diego, CA, USA; Melinta Therapeutics, LLC, Parsippany, NJ, USA; Cidara Therapeutics, Inc., San Diego, CA, USA; Melinta Therapeutics, LLC, Parsippany, NJ, USA; University of Alabama at Birmingham, Birmingham, AL, USA; JMI Laboratories, North Liberty, IA, USA; University of California Davis Medical Center, Sacramento, CA, USA

## Abstract

The increasing prevalence of candidemia and invasive candidiasis infections caused by *Candida auris* represents a global health risk. Such infections are difficult to treat as they are often multidrug-resistant and are linked to high rates of mortality. Rezafungin is a second-generation echinocandin with antifungal activity against a range of *Candida* species, including wild type, and azole- and some echinocandin-resistant isolates. Its stability and prolonged half-life permit less frequent dosing compared with other echinocandins, leading to high front-loaded exposures and potential earlier mycological clearance from infection sites. These properties make rezafungin a candidate for the treatment of candidemia and invasive candidiasis infections due to *C. auris*. Accordingly, this narrative review article describes available evidence for the activity and effectiveness of rezafungin against *C. auris* isolates and infections. To date, the activity of rezafungin against *C. auris* isolates and infections has been demonstrated in *in vitro* and *in vivo* non-clinical experiments, and in pharmacokinetic/pharmacodynamic target attainment estimations utilizing clinical data. With similar potency to other echinocandins, rezafungin demonstrates *in vitro* and *in vivo* activity that is comparable to or better than that seen with other echinocandins, and similar to that for rezafungin in other *Candida* species. Like other echinocandins, its activity is reduced in *fks*-mutant isolates. Although there is currently a dearth of data on the therapeutic activity of rezafungin against *C. auris*, it is reasonable that rezafungin may be a viable choice for treating candidemia and invasive candidiasis caused by *C. auris*. Further clinical investigations are necessary.

## Introduction

Despite widespread availability of antifungal medicines, candidemia and invasive candidiasis (IC) remain major causes of morbidity and mortality in the healthcare setting.^1,2^  *Candida albicans* is the most common causative organism, but infections due to non-*albicans Candida* species are increasing and there has been a concomitant rise in reports of antifungal resistance, including multidrug resistance.^1–6^ These changes are likely to be partly due to the abundant use of antifungals in the healthcare environment.^7^ Though the recent increase in antifungal resistance is well documented, the true prevalence of resistance, especially multidrug resistance, is difficult to quantify due to differences in resistance among species and geographic regions.^3,8,9^

*Candida auris*, which causes candidemia and other invasive infections, is classified as a critical priority fungal pathogen by the WHO and poses a particularly serious global health threat.^3,6,10–18^ Such infections are notoriously difficult to treat as they are frequently non-susceptible to multiple antifungal agents, including FLC, AMB and/or echinocandins, and consequently are associated with high mortality, with rates ranging from 29% to 53%.^3,6,11,15,18–20^ Infection control concerns are compounded by the ineffectiveness of quaternary ammonia compounds in eradicating environmental *C. auris*.^21^ Similar to *Candida glabrata*, the predominantly haploid state of *C. auris* predisposes this species to acquiring mutation-based antifungal drug resistance.^22^

*C. auris* infections are easily transmitted owing to environmental persistence and skin colonization, with fungal isolates able to adhere to and survive on skin, indwelling devices, and abiotic surfaces for prolonged periods even after stringent cleaning practices are employed.^3,6,7,11^ The virulence and transmissibility of *C. auris* are facilitated by the genetic diversity of this species and its ability to produce adhesins, which enable it to adhere to host tissues and medical devices, and to form biofilms, undergo morphological transformation, produce hydrolytic enzymes and employ a range of strategies to evade the host immune response.^7^

**Figure 1.**
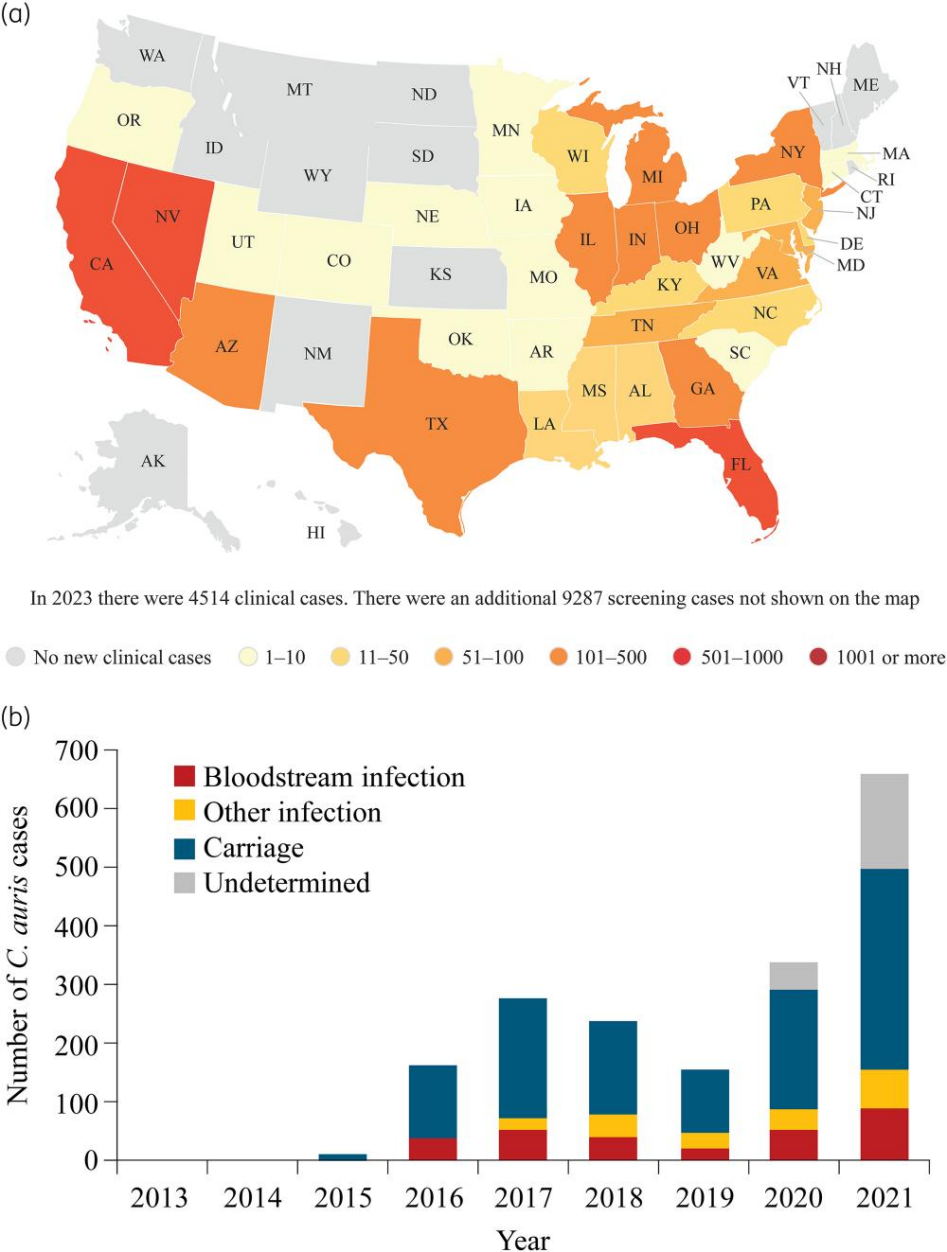
(*A*) Clinical cases of *C. auris* reported in the USA (2023; *n *= 4514);^16^ (*B*) Case reports of *C. auris* infection or carriage within countries included in the EU or EEA, excluding the UK (2013–21; *n *= 1812).^12^ AK, Alaska; AL, Alabama; AR, Arkansas; AZ, Arizona; CA, California; CO, Colorado; CT, Connecticut; DE, Delaware; EEA, European Economic Area; EU, European Union; FL, Florida; GA, Georgia; HI, Hawaii; IA, Iowa; ID, Idaho; IL, Illinois; IN, Indiana; KS, Kansas; KY, Kentucky; LA, Louisiana; MA, Massachusetts; MD, Maryland; ME, Maine; MI, Michigan; MN, Minnesota; MO, Missouri; MS, Mississippi; MT, Montana; NC, North Carolina; ND, North Dakota; NE, Nebraska; NH, New Hampshire; NJ, New Jersey; NM, New Mexico; NV, Nevada; NY, New York; OH, Ohio; OK, Oklahoma; OR, Oregon; PA, Pennsylvania; RI, Rhode Island; SC, South Carolina; SD, South Dakota; TN, Tennessee; TX, Texas; UT, Utah; VA, Virginia; VT, Vermont; WA, Washington; WI, Wisconsin; WV, West Virginia; WY, Wyoming. Panel (*A*) was developed by the US CDC. This reuse does not constitute endorsement or recommendation by the US Government, Department of Health and Human Services, or CDC. This material is freely available on the agency website at: https://www.cdc.gov/candida-auris/tracking-c-auris/?CDC_AAref_Val=https://www.cdc.gov/fungal/candida-auris/tracking-c-auris.html. Panel (*B*) was reproduced with minor modifications from Kohlenberg *et al.*^12^ This is an open-access article distributed under the terms of the Creative Commons Attribution 4.0 International License.

Infections due to *C. auris* are still relatively uncommon in many regions compared with invasive infections caused by other *Candida* species, but the number of *C. auris* infections has risen sharply over time since the first clinical case report in Japan in 2009; noting that a very early case has since been traced back retrospectively to 1996 in a patient with candidemia from South Korea.^3,12,16,18,23–25^ In 2023, 4514 clinical cases of *C. auris* were reported to the US CDC (*Figure 1A*),^16^ while across the European Union (EU) and European Economic Area, 990 cases of *C. auris* were reported in 2020–21 (*Figure 1B*).^12^ Actual rates are likely to be higher, however, as *C. auris* infections are often misidentified or misdiagnosed, owing to inadequate diagnostics and/or identification strategies, or not reported at all as notification is not mandatory in many countries.^3,7,20^

Whole genome sequencing has revealed that *C. auris* can be separated into four main distinct genetic clades with different geographic distributions: Clade I, the South Asian clade (the most prevalent); Clade II, the East Asian clade; Clade III, the South African clade; and Clade IV, the South American clade.^3,18,19^ Two additional new clades have recently been discovered in Iran (Clade V), and Bangladesh and Singapore (Clade VI).^26–28^ The different clades seem to have different antifungal-susceptibility profiles, with Clade I being the least susceptible to all the available treatments and Clade II the most susceptible.^3,29^ Clinical susceptibility breakpoints for antifungals, based on MICs, have yet to be established for *C. auris* by USA and European organizations for antimicrobial susceptibility testing (the CLSI and EUCAST, respectively). The only exception to date is a susceptible-only CLSI breakpoint for rezafungin against *C. auris*.^30^ Nevertheless, tentative breakpoints have been proposed by the US CDC for the most commonly used antifungal agents, based on those established for closely related *Candida* species and on expert opinion (Table 1).^31^ These breakpoints indicate that ∼90% of *C. auris* isolates in the USA can be classified as resistant to FLC, ∼30% as resistant to AMB and <2% as resistant to echinocandins.^31^

**Table 1.** CDC tentative breakpoints for antifungal susceptibility to *C. auris*^31^

Antifungal agent	Tentative MIC breakpoint (mg/L)^a^	Comments
FLC	≥32	Modal MIC was ≥256; isolates with MICs ≥32 had a resistance mutation in the *Erg11* gene, making them unlikely to respond to FLC
VRC and other second-generation triazoles	N/A	Consider using FLC susceptibility as a surrogate for susceptibility. However, isolates that are resistant to FLC may occasionally respond to other triazoles; decisions to treat with another triazole should be made on a case-by-case basis
AMB	≥2	Recent PK/PD analysis of *C. auris* in a mouse model of infection indicates that, under standard dosing, breakpoint should be 1 or 1.5. Therefore, isolates with MIC ≥2 should be considered resistant. If using Etest and MIC is determined as 1.5, the value to be rounded-up to 2
Anidulafungin	≥4	Tentative breakpoints based on modal distribution of echinocandin MICs of ∼100 isolates from diverse geographic locations
CAS	≥2	—
Micafungin	≥4	—

Table developed by the US CDC. This reuse does not constitute endorsement or recommendation by the US Government, Department of Health and Human Services, or CDC. This material is freely available on the agency website at: https://www.cdc.gov/candida-auris/hcp/laboratories/antifungal-susceptibility-testing.html#.

^a^At the time of these guidelines, there were no established *C. auris*-specific susceptibility breakpoints; therefore, these tentative breakpoints were defined based on those for closely related *Candida* species and on expert opinion. These tentative breakpoints are based on antifungal-susceptibility tests that follow CLSI guidelines.

MIC, minimum inhibitory concentration; N/A, not available; PD, pharmacodynamic; PK, pharmacokinetic.

Owing to their efficacy against the range of *Candida* species and low probability of adverse events, the recommended first-line treatments for patients with candidemia and/or IC infections are echinocandins, with FLC and liposomal AMB acceptable alternatives.^32,33^ However, because of the emergence of resistant causative pathogens, including multidrug-resistant *C. auris*, new treatment options are needed. Drugs in late-stage clinical development or recently approved for the treatment of candidemia/IC include the new echinocandin rezafungin, the novel Gwt1 enzyme inhibitor fosmanogepix and the glucan synthase inhibitor ibrexafungerp.^34^ These may have potential in treating drug-resistant infections such as those due to *C. auris*.

**Figure 2.**
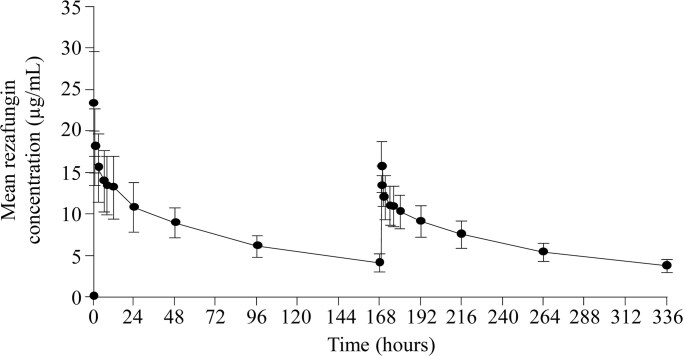
Pharmacokinetics of a 400 mg dose of rezafungin followed by a 200 mg dose once-weekly. Figure reproduced from Andes *et al.*, by permission of Oxford University Press.^50^

Rezafungin, which is the focus of this review, is a second-generation echinocandin that has shown activity against a range of *Candida* species, including wild type, azole- and subsets of echinocandin-resistant isolates.^35–45^ It has improved chemical and biologic properties that differentiate it from other echinocandins. Most notably, rezafungin’s stable chemical structure, with reduced metabolism and degradation relative to other echinocandins, results in a prolonged half-life, allowing once-weekly dosing^46–49^ (*Figure 2*^50^). In turn, less frequent dosing results in high front-loaded exposures, which along with good distribution to and penetration at infection sites, may enable earlier mycological clearance from the sites of infection.^39,47,48,51–55^ As such, rezafungin could potentially reduce the risk of resistance development.

Rezafungin was first approved in 2023 in the USA, and is now approved in multiple countries (including the EU), as a treatment for candidemia and/or IC in adults, with *in vitro* and clinical activity demonstrated against most *C. albicans*, *C. glabrata*, *Candida parapsilosis*, and *Candida tropicalis* isolates.^56,57^ These approvals were based on clinical efficacy and safety data from patients with candidemia and/or IC from the randomized Phase 2 STRIVE and Phase 3 ReSTORE trials.^51,53^ In the Phase 3 ReSTORE trial, which included patients with infections caused by various *Candida* species (including *C. albicans*, *C. glabrata*, *C. parapsilosis*, and *C. tropicalis*, but not *C. auris*), rezafungin showed non-inferior efficacy to CAS for global cure at Day 14 and all-cause mortality at Day 30.^51^ The clinical data also suggested potentially faster rates of mycological eradication with rezafungin compared with CAS.^51–53^

Given the lack of *C. auris* infections in the registrational clinical trials, the objective of this review article was to identify and describe the available evidence for the activity and effectiveness of rezafungin against *C. auris*.

## Literature search strategy

Literature searches were conducted using PubMed to capture data from microbiology publications, preclinical studies, pharmacokinetic/pharmacodynamic (PK/PD) analyses, observational studies, case reports, and clinical studies published up to 16 January 2025 (in any language), on the activity and efficacy/effectiveness of rezafungin against *C. auris.* The search terms used were ‘rezafungin’, ‘CD101’ and ‘*Candida auris’*. Results identified for inclusion comprised microbiology publications, *in vivo* preclinical data, and PK/PD analyses. Recent congress abstracts (2022–present) were searched using the same terms. Abstract databases that were interrogated included those for meetings of ESCMID, formerly the European Congress of Clinical Microbiology and Infectious Diseases (ECCMID); European Society of Intensive Care Medicine (ESICM), International Immunocompromised Host Society (ICHS), IDWeek [Annual Meeting of the IDSA, Society for Healthcare Epidemiology of America (SHEA), HIV Medicine Association (HIVMA), Pediatric Infectious Diseases Society (PIDS), and Society of Infectious Diseases Pharmacists (SIDP)], International Symposium on Intensive Care and Emergency Medicine (ISICEM), and Trends in Medical Mycology (TIMM).

Search results were reviewed manually to identify references for inclusion based on their relevance to the topic of rezafungin and *C. auris*, in the opinion of the authors. Additional references could be included from the bibliographies of articles identified in the primary search or based on the knowledge of the authors. The search results were also supplemented with manufacturer submissions to the CLSI and EUCAST.

## Literature search results

The literature search identified eight microbiology studies (six publications and two congress presentations), two *in vivo* preclinical studies (one publication and one presentation) and three PK/PD studies (two full publications plus a submission to EUCAST), the results from which are described. No clinical trials, observational studies, or case reports describing the efficacy or effectiveness of rezafungin against infections caused by *C. auris* were identified.

## *In vitro* susceptibility studies

The key findings from the eight identified microbiology studies describing the *in vitro* susceptibility of rezafungin against *C. auris* isolates are summarized in Table 2. In the first study, the *in vitro* activity of rezafungin against 100 *C. auris* isolates from the four established clades (countries of origin: Pakistan, Venezuela, Panama, Colombia, India, South Africa, Israel and the USA) was tested using CLSI M27 reference-standard broth microdilution methodology.^35,64,65^ Rezafungin showed potent activity against most *C. auris* isolates, including some that were resistant to other echinocandins. MIC values for rezafungin ranged from 0.03 to 8 mg/L. In comparison, the range of MIC values for other echinocandins was 1 to >16 mg/L for anidulafungin, 0.5 to >16 mg/L for CAS and 0.5 to >8 mg/L for micafungin. Importantly, the MIC values for rezafungin in *C. auris* were consistent with those determined for other *Candida* species in separate *in vitro* studies,^36–38,41–45^ and there were no apparent differences in susceptibility to rezafungin among the four *C. auris* clades, indicating a lack of impact of genetic diversity on the activity of this antifungal. Among eight isolates with elevated MICs to one or more of anidulafungin, CAS or micafungin, MIC values for rezafungin ranged from 0.06 to 8 mg/L, with an MIC_50_ of 0.5 mg/L. Four isolates possessing the Fks1 hotspot 1 (HS1) S639P substitution had the highest MICs to rezafungin (4 or 8 mg/L), and had similarly elevated MIC values to the other echinocandins, consistent with the impact of target-based *FKS* mutations on this drug class in other *Candida* species.^35^

**Table 2.** *In vitro* susceptibility of *C. auris* isolates to rezafungin and comparator antifungals

Study	Broth microdilution testing method	*C. auris* isolates, *n* (*fks1* mutants, *n*)	Antifungal		MIC values (mg/L)	
				Range	MIC_50_	MIC_90_
Berkow & Lockhart^35^	CLSI M27-A3	100 (4)	Rezafungin	0.03 to 8	0.125	0.5
			Anidulafungin	1 to >16	NR	NR
			CAS	0.5 to >16	NR	NR
			Micafungin	0.5 to >8	NR	NR
Helleberg *et al.*^38^	EUCAST E.Def 7.3.1^b^	122 (8)	Rezafungin	0.06 to 16	0.25	1
			Anidulafungin	0.016 to >32	0.125	NR
			Micafungin	0.03 to >32	0.125	NR
			FLC	0.5 to >64	≥64	NR
			VRC	≤0.004 to 4	0.5	NR
			Isavuconazole	≤0.004 to 2	0.125	NR
			AMB	0.5 to 1	1	NR
Tóth *et al.*^58^	CLSI M27-Ed4	19 (NR)	Rezafungin	0.03 to 0.25	0.12^a^	0.25^a^
			Anidulafungin	0.03 to 0.5	0.06^a^	0.25^a^
			CAS	0.25 to 1	0.5^a^	1^a^
			Micafungin	0.06 to 2	0.25^a^	0.5^a^
			FLC	0.5 to >32	>32^a^	>32^a^
			AMB	0.12 to 1	0.5^a^	1^a^
Kovács *et al.*^59^	CLSI M27-Ed4	13 (NR)	Rezafungin	0.03 to 0.25	NR	NR
			Anidulafungin	0.015 to 0.25	NR	NR
			CAS	0.12 to 1	NR	NR
			Micafungin	0.03 to 0.25	NR	NR
Larkin *et al.*^60^	CLSI M27-A3	16 (NR)	Rezafungin	0.031 to 1	0.125^a^	0.25^a^
			Anidulafungin	0.125 to 0.25	0.125^a^	0.25^a^
			CAS	0.25 to 1	0.5^a^	1^a^
			Micafungin	0.25 to 2	1^a^	1^a^
			FLC	1 to >64	16^a^	>64^a^
			VRC	<0.063 to 1	0.5^a^	1^a^
			Itraconazole	<0.063 to 1	0.5^a^	1^a^
			AMB	0.5 to 8	2^a^	4^a^
Carvalhaes *et al.*^61^	CLSI M27-Ed4	9 (1)	Rezafungin	0.25 to >4	0.5^a^	NR
			Anidulafungin	0.25 to 4	0.5^a^	NR
			CAS	0.06 to >4	0.12^a^	NR
			Micafungin	0.12 to 4	0.25^a^	NR
			FLC	NR	>128^a^	NR
			VRC	NR	1^a^	NR
Castanheira *et al.*^24^	CLSI M27-Ed4	78 (2)	Rezafungin	0.008 to >4	0.25 (Clades I, IV), 0.5 (Clade III)^c^	0.5
			Anidulafungin	0.06 to 4	0.25 (Clades I, IV), 0.5 (Clade III)^c^	0.5
			CAS	0.015 to >4	0.12 (Clade I), 0.06 (Clades III, IV)^c^	0.25
			Micafungin	0.06 to 4	0.12	0.25
			FLC	2 to >128	128 (Clade I), > 128 (Clade III), 64 (Clade IV)^c^	>128 (Clade I), 128 (Clade IV)^c^
			AMB	0.5 to 4	1 (Clades I and IV), 0.5 (Clade III)^c^	2 (Clade I), 1 (Clade IV)^c^
Winkler *et al.*^62^	CLSI M27-Ed4	65 (NR)	Rezafungin	0.06 to >4	0.25	0.5
			Anidulafungin	0.12 to 4	0.25	0.5
			CAS	0.015 to >4	0.12	0.25
			Micafungin	0.06 to 4	0.12	0.25
			FLC	2 to >128	128	>128
			VRC	0.015 to 4	0.5	2
			AMB	0.5 to 4	1	2

MIC_50_, MIC values at which growth was inhibited in 50% of isolates; MIC_90_, MIC values at which growth was inhibited in 90% of isolates; *n*, number of isolates; NR, not reported.

^a^MIC_50_/MIC_90_ estimates must be interpreted with caution due to the limited number of isolates.

^b^The EUCAST E.Def 7.3.1 testing methodology has since been shown to be incompatible with susceptibility testing for rezafungin due to unacceptable inter-laboratory MIC variation for *Candida* species with lower echinocandin MIC values, and should be viewed in the context of that limitation.^63^

^c^Clade I: South Asia (*n *= 40), Clade II: East Asia (*n *= 1), Clade III: South Africa (*n *= 7), Clade IV: South America (*n *= 30).

Rezafungin also demonstrated *in vitro* activity against *C. auris* in a study of 122 isolates from India, of which 15% were classified as non-wild type (Table 2).^38^ Although the MIC values were similar to those reported by Berkow & Lockhart using CLSI M27 reference standards,^35^ they were determined using EUCAST E.Def 7.3.1 testing methodology,^66^ which has since been shown to be incompatible with susceptibility testing for rezafungin due to unacceptable inter-laboratory MIC variation for *Candida* species with lower echinocandin MIC values, and should be viewed in the context of that limitation.^63^ The EUCAST methodology for rezafungin testing has now been replaced with EUCAST E.Def 7.4 broth microdilution methodology that specifies the use of RPMI-1640 assay medium containing 0.002% Tween 20.^67–69^ Consistent with Berkow & Lockhart,^35^ susceptibility to rezafungin was substantially reduced for the eight isolates in this study that had *FKS* HS mutations (MIC values of 8 or 16 mg/L); these *fks*-mutant isolates were also resistant to other echinocandins and FLC, but were susceptible to AMB.

Four additional susceptibility studies, all using CLSI M27 broth microdilution methodology, have shown similar levels of antifungal activity for rezafungin against *C. auris*.^58–61^ In one study of 19 *C. auris* isolates, MIC values for rezafungin ranged from 0.03 to 0.25 mg/L.^58^ For other echinocandins tested in this study, none of the MIC values for *C. auris* exceeded the tentative breakpoints proposed by the CDC (Table 1). The MIC_50_ and MIC_90_ values indicated that the potency of rezafungin was similar to that of anidulafungin, but greater than that of micafungin and CAS (Table 2). However, the MIC_50_ and MIC_90_ estimates must be interpreted with caution due to the limited number of isolates.

In a smaller study of 13 *C. auris* isolates from four clades (South Asian *n *= 3, East Asian *n *= 3, South American *n *= 4 and South African *n *= 3), MIC values for rezafungin ranged from 0.03 to 0.25 mg/L (Table 2).^59^ Here, rezafungin showed the same or greater activity at clinically obtainable trough concentrations compared with anidulafungin, CAS and micafungin against all four clades.

Another *in vitro* study reported MIC values for rezafungin of 0.03 to 1 mg/L against 16 *C. auris* isolates obtained from patients in Germany, India, Japan and South Korea between 2009 and 2016 (Table 2).^60^ Although the limited number of isolates prevents reliable comparisons, the rezafungin MIC_50_ and MIC_90_ tentative estimates showed similar potency to anidulafungin, but 4-fold greater potency than micafungin and CAS. Eight of the 16 isolates had susceptibility profiles that, for other *Candida* species, would be assessed as being resistant to at least three of the antifungals tested: anidulafungin, micafungin, CAS, FLC, itraconazole, VRC or AMB.

Three MIC studies described evaluated data from the 2022 SENTRY Rezafungin Surveillance Program.^24,61^ In the first study, rezafungin was tested against nine FLC-resistant *C. auris* isolates: four from Europe, three from North America and two from Latin America.^61^ Seven of these isolates were susceptible to rezafungin according to the CLSI-susceptible only criteria of ≤0.5 mg/L (77.8% susceptible; MIC values of 0.25 or 0.5 mg/L). One of the two non-susceptible isolates (rezafungin MIC >4 mg/L; origin, Panama) was resistant to all echinocandins (MIC ≥4 mg/L) and carried the R1354G alteration (Fks1 HS2). Eight of the nine *C. auris* isolates were susceptible to anidulafungin, CAS and micafungin (88.9% susceptible) per their tentative CDC breakpoints (breakpoints: MIC ≥2 mg/L for CAS and ≥4 mg/L for anidulafungin and micafungin).

A second study from the 2022 SENTRY Antifungal Surveillance Program described the *in vitro* activity of rezafungin and other antifungals against 78 *C. auris* isolates.^24^ Tested using CLSI broth microdilution methodology, MIC values for rezafungin of 0.008 to >4 mg/L were detected across the four main clades of *C. auris* (Table 2). Based on a recently adopted susceptible CLSI breakpoint of 0.5 mg/L,^30^ 96.2% of *C. auris* isolates were deemed susceptible to rezafungin. Susceptibility to rezafungin was 100% in the South Asian clade (*n *= 40; MIC range, 0.06 to 0.5 mg/L; MIC_50_/MIC_90_, 0.25/0.5 mg/L), 96.7% in the South American clade (*n *= 30; MIC range, 0.03 to >4 mg/L; MIC_50_/MIC_90_, 0.25/0.5 mg/L) and 71.4% in the South African clade (*n *= 7; MIC range, 0.125 to 1 mg/L; MIC_50_ 0.5 mg/L). The single isolate in the East Asian clade was also susceptible to rezafungin (MIC, 0.008 mg/L). Notably, according to the MIC_50_ and MIC_90_ estimates, rezafungin showed similar activity to anidulafungin, but was marginally less potent than micafungin and CAS. Most of the 78 *C. auris* isolates (82.1%) were resistant to FLC, with almost one in five (17.9%) resistant to AMB.

Lastly, a third study from the SENTRY Program reported on the *in vitro* activity of rezafungin against 28 uncommon species of *Candida* collected between 2020 and 2022.^62^ Here, rezafungin was tested at concentrations ranging from 0.002 to 4 mg/L, using the CLSI broth microdilution method, against 65 *C. auris* isolates collected from various regions of the world (Asia-Pacific *n *= 1, Europe *n *= 32, Latin America *n *= 16 and North America *n *= 16). MIC values for rezafungin in *C. auris* ranged from 0.06 to >4 mg/L (MIC_50_/MIC_90_, 0.25/0.5 mg/L) (Table 2). Based on the susceptible CLSI breakpoint of 0.5 mg/L,^30^ 95.4% of *C. auris* isolates were susceptible to rezafungin. Considering MIC_50_ and MIC_90_ estimates, rezafungin showed comparable activity to anidulafungin, but was slightly less potent than CAS and micafungin. The *C. auris* isolates were 98.5% non-resistant to all three other echinocandins based on CDC resistant-only breakpoint definitions. In contrast, just 10.8% of *C. auris* were non-resistant to FLC (MIC range, 2 to >128 mg/L) and 84.6% were non-resistant to AMB (MIC range, 0.5 to 4 mg/L) according to the CDC resistant breakpoints. As shown for *C. auris*, rezafungin was within 2-fold of the MIC_50_/MIC_90_ values for the other three echinocandins against all 28 uncommon *Candida* species tested.

Taken together, these studies indicate the potent *in vitro* activity of rezafungin against *C. auris* regardless of clade. Available data suggest that rezafungin has comparable potency to other echinocandins, and its activity against *C. auris* is similar to that seen against other *Candida* species.^36–38,41–45^ As also reported with other *Candida* species,^36–38,41–45^ the activity of rezafungin against *C. auris* is impacted by the presence of *FKS* gene mutations, which represents the predominant mechanism of resistance to echinocandins.^70^

## *In vitro* time-kill kinetics

The time–kill kinetics of rezafungin were compared with those of anidulafungin, CAS and micafungin in the aforementioned *in vitro* study of 13 *C. auris* isolates from the four main geographic clades.^59^ Compared with other licensed echinocandins, rezafungin showed the same or greater killing activity against all four of the *C. auris* clades at concentrations considered to be clinically obtainable (dose range tested, 0.25 to 32 mg/L). All four antifungals were fungistatic (defined as a <99.9% reduction in viable cell count versus the starting inoculum); inhibition of isolates was generally only seen during the first 8–12 h, with prominent regrowth after 24 h. When tested in RPMI-1640 medium without human serum, none of the four echinocandins dosed up to 32 mg/L killed any isolates from the South African clade, nor two isolates from the South American clade, after 24 h. Against the East Asian and South Asian clades, only rezafungin and anidulafungin (at ≥4 to ≥8 mg/L, and ≥8 to ≥16 mg/L, respectively) achieved the threshold for killing after 24 h. However, when tested in RPMI-1640 medium with 50% human serum, rezafungin showed killing activity across all four clades, and was the only echinocandin to do so in the South African clade. As rezafungin produces high *in vivo* exposures from the start of treatment in human subjects [mean maximum concentration of up to 22.7 mg/L in plasma following a single 400 mg dose (2.6- to 4.0-fold higher than can be achieved with clinical doses of other licensed echinocandins)], with notable levels even after 48–72 h,^48^ the authors postulated that the drug should enable early improved killing and a prolonged therapeutic effect to maintain elimination of *C. auris*, including isolates from the South American and South African clades, against which other echinocandins appear to be less active.

## *In vivo* models of IC

**Figure 3.**
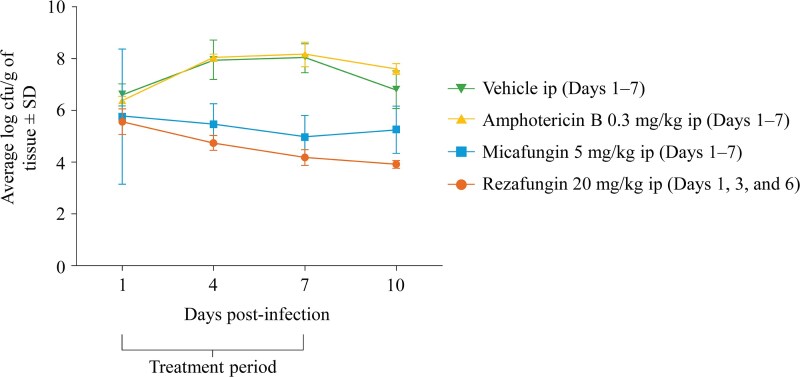
Efficacy of rezafungin for the treatment of disseminated *C. auris* infection in an immunocompromised mouse model: average log cfu/g in kidney tissue over time. cfu, colony-forming units; ip, intraperitoneally; SD, standard deviation. Figure reproduced from Hager *et al.*, by permission of Oxford University Press.^71^

Two preclinical studies conducted in *in vivo* mouse models have evaluated the antifungal efficacy of rezafungin in the treatment of *C. auris* infections.^71,72^ In the first of these studies, the efficacy of rezafungin in the treatment of disseminated *C. auris* infection was explored using a mouse model of disseminated candidiasis.^71^ For this randomized study, immunosuppressed mice (*n *= 20 in each group) received rezafungin 20 mg/kg, AMB 0.3 mg/kg, micafungin 5 mg/kg, or vehicle 2 h after being infected with *C. auris*. Two further doses of rezafungin were given on Days 3 and 6, while the remaining groups were treated daily for a total of seven doses. Five animals from each group were sacrificed on Days 1, 4, 7, and 10, and the kidneys removed to determine the effect of antifungal treatment on kidney tissue fungal burden. Rezafungin was shown to be more effective than both micafungin and AMB (*Figure 3*). Treatment with rezafungin was associated with a significantly lower average log_10_ cfu/g of tissue compared with AMB and vehicle at all time points. In addition, rezafungin-treated mice had significantly lower average log_10_ cfu/g of tissue on Day 10 compared with mice treated with micafungin. These results suggest that, while rezafungin is dosed less frequently than AMB or micafungin, it is more efficacious in reducing *C. auris* fungal burden in this setting. This finding could be due to the distinct PK/PD profile of rezafungin that enables front-loaded dosing and high initial therapeutic exposures.^46–48,73,74^

In a second study, the *in vivo* efficacy of rezafungin, anidulafungin, CAS and micafungin were compared against four *C. auris* clades in a neutropenic mouse infection model.^72^ Ten isolates representing the four *C. auris* clades (South Asian, *n *= 2; East Asian, *n *= 2; South African *n *= 2; South American *n *= 4) were utilized. Neutropenia was induced prior to infection and maintained throughout the course of the experiments. The neutropenic mice (*n *= 5 per treatment group) were infected with *C. auris*, and 24 h afterwards treated with rezafungin 20 mg/kg on Days 1, 3 and 6, or once-daily CAS 3 mg/kg, micafungin 5 mg/kg, or anidulafungin 5 mg/kg (all clinically equivalent doses) on Days 1–6. Fungal tissue burden was assessed in kidneys, hearts and brains on Day 7. Rezafungin demonstrated efficacy that was comparable to or better than anidulafungin, CAS and micafungin against all four *C. auris* clades. All the active treatments generally produced >3-log mean reductions in fungal kidney and heart burden, although some decreases were not statistically significant. Irrespective of the clade, rezafungin produced 3- to 5-log cfu decreases in kidneys and 2- to 4-log cfu decreases in hearts. None of the tested drugs inhibited fungal growth in the brain, indicating a lack of activity against CNS infections. Rezafungin has been shown previously to have limited CNS penetration,^47^ consistent with other members of the echinocandin class.^75–77^ In separate investigations (*n *= 10 mice per group), all echinocandins improved survival at Day 7 irrespective of the isolates and clades.^72^

Considering the results of both studies, rezafungin appears to be efficacious against *C. auris* in *in vivo* models of candidiasis. The activity of rezafungin against *C. auris* infections was comparable to or better than that seen with other echinocandins. Similar potent *in vivo* activity has been demonstrated for rezafungin against other *Candida* species in murine models of disseminated candidiasis.^39,40,78^

## PK/PD evaluations

One PK/PD study reported exposures and PD activity for rezafungin in a neutropenic mouse model of IC against four *C. auris* strains, with rezafungin CLSI-defined MIC values ranging from 0.06 to 2 mg/L.^74^ Neutropenic mice (*n *= 3 per group) were inoculated with *C. auris* and 2 h later treated with rezafungin at doses of 1, 4, 16, or 64 mg/kg, followed by two additional doses on Days 3 and 6. On Day 7, animals were sacrificed for determination of fungal colony-forming units in the kidneys. PK exposures were obtained locally, with protein binding of 99.2% (in mouse plasma) used to determine free-drug concentrations. Susceptibility testing was based on CLSI guidelines. As shown in similar experiments (described above),^71,72^ rezafungin showed potent *in vivo* activity against *C. auris*. A 1-log kill was achieved for three of the four strains; only the most resistant strain harbouring an *FKS1* HS1 mutation (S639F substitution; MIC 2 mg/L) did not achieve a 1-log-kill endpoint over the dose range tested. As demonstrated in other *Candida* species,^39,40^ the index area under the plasma concentration–time curve (AUC)/MIC ratio for rezafungin strongly predicted efficacy. Across all isolates, the stasis and 1-log-kill free-drug (*f*) AUC_0–24h_/MIC targets were determined to be 1.88 and 5.77, respectively. These non-clinical PK/PD targets are comparable to those reported for other *Candida* species in similar *in vivo* models.^39,40^

**Figure 4.**
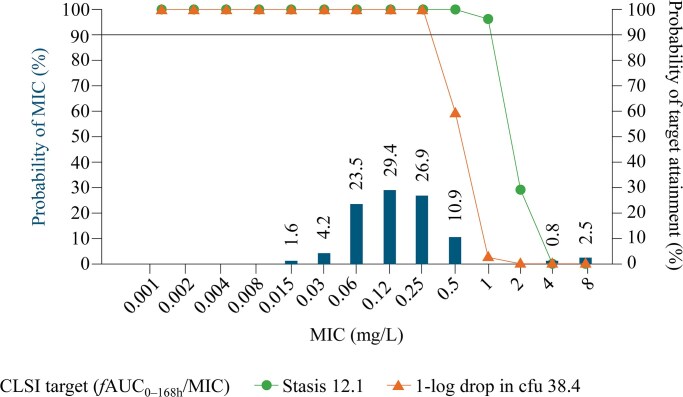
Probability of PK/PD target attainment for rezafungin 400 mg against *C. auris* using CLSI methodology.^79^  *f*AUC_0–168 h_, free-drug area under the plasma concentration–time curve from 0 to 168 h; cfu, colony-forming units; CLSI, Clinical and Laboratory Standards Institute; MIC, minimum inhibitory concentration. This figure shows the estimated PK/PD target attainment for net fungal stasis and 1-log_10_ drop in colony-forming units using *f*AUC_0–168 h_ values following a 400 mg loading dose of rezafungin. CLSI methodology was used to calculate MICs over the MIC distribution for *C. auris.* The bars and associated data labels represent the observed distribution of MIC values in the surveillance data. The horizontal line represents the simulated 90% probability of CLSI target attainment, i.e. 90% probability of achieving a *f*AUC_0–168 h_/MIC target of 12.1 for net fungal stasis and 38.4 for 1-log_10_ drop in colony-forming units. The lines with circles and triangles indicate the per cent probability of CLSI target attainment for stasis and 1-log_10_ drop in colony-forming units, respectively, at each CLSI-defined MIC value. Protein binding of 97.4% was assumed from healthy subject data. The simulated probabilities of target attainment for both targets (net fungal stasis and 1-log_10_ drop in colony-forming units) were >90% at MIC values of ≤0.25 mg/L. For stasis alone, the simulated probability of target attainment was >90% at MIC values of ≤1 mg/L. Figure reproduced with minor modifications from Roepcke *et al*.^79^ This is an open-access article distributed under the terms of the Creative Commons Attribution 4.0 International License.

A subsequent population PK model was developed using data from five Phase 1 trials and the Phase 2 STRIVE and Phase 3 ReSTORE trials.^79^ This analysis performed target attainment simulations to estimate the probability of achieving PK/PD targets across a range of MIC values (0.001 to 8 mg/L), using CLSI broth microdilution methods, for *C. auris* and five other *Candida* species.^79^ The likelihood of achieving non-clinical PK/PD targets (as defined by Lepak *et al.*^74^) associated with net fungal stasis (*f*AUC_0–168h_/MIC ratio, 12.1) and a 1-log_10_ drop in colony-forming units from baseline (*f*AUC_0–168 h_/MIC ratio, 38.4) for *C. auris* after a single 400 mg rezafungin loading dose was determined using simulated *f*AUC_0–168h_/MIC ratios across 100 datasets, each comprising 1000 virtual patients. Protein binding of 97.4% (in human plasma) was used to adjust for unbound *f*AUC_0–168 h_ and to determine separate *f*AUC_0–168 h_/MIC ratio variables for the range of MIC values. The MIC susceptibility breakpoint was determined as the highest clinically relevant MIC value with a ≥90% probability of PK/PD target attainment. For *C. auris*, the simulations indicated that probability of target attainment following a single 400 mg dose of rezafungin was >90% for both targets (net fungal stasis and 1-log_10_ cfu reductions) at MIC values of ≤0.25 mg/L (based on CLSI determination) (*Figure 4*). For stasis alone, the probability of target attainment was ≥90% at MIC values of ≤1 mg/L. Rezafungin was also predicted to have a high probability of target attainment in other *Candida* species, covering the majority of the observed MIC distributions for most organisms tested.^79^

**Figure 5.**
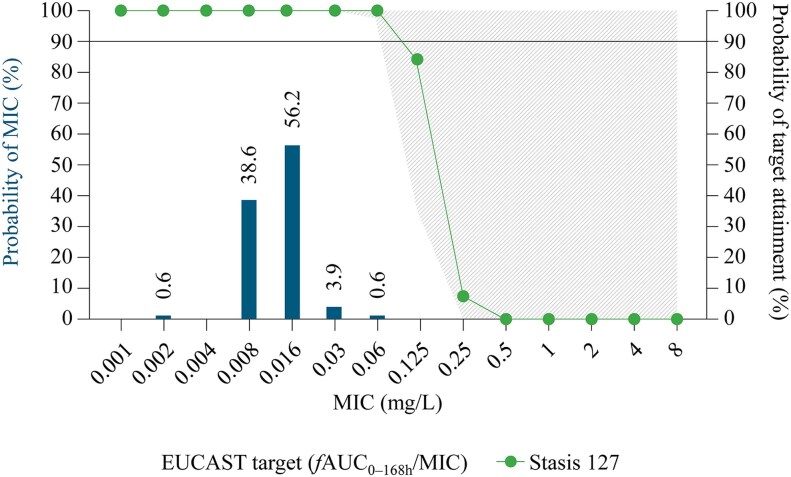
Probability of PK/PD target attainment for rezafungin 400 mg against *C. auris* using EUCAST E.Def 7.4 methodology with RPMI-1640 assay medium containing 0.002% Tween 20.^80^  *f*AUC_0–168 h_, free-drug area under the plasma concentration–time curve from 0 to 168 h; EUCAST, European Committee on Antimicrobial Susceptibility Testing; MIC, minimum inhibitory concentration. This figure shows the estimated PK/PD target attainment for net fungal stasis using *f*AUC_0–168 h_ values following a 400 mg loading dose of rezafungin. EUCAST methodology was used to calculate MICs over the MIC distribution for *C. auris.* The bars and associated data labels represent the observed distribution of MIC values in the surveillance data. The horizontal line represents the simulated 90% probability of EUCAST target attainment, i.e. 90% probability of achieving a *f*AUC_0–168 h_/MIC target of 127 for net fungal stasis. The line with circle symbols indicates the per cent probability of EUCAST target attainment for stasis at each EUCAST-defined MIC value. Protein binding of 97.4% was assumed from healthy subject data. The simulated probability of target attainment for net fungal stasis was ≥95% at MIC values of ≤0.06 mg/L. Figure reproduced with minor modifications from EUCAST. REZAFUNGIN: rationale for the clinical breakpoints, version 1.0 2023. These data have been produced in part under the European Centre for Disease Prevention and Control service contracts, is made available at no cost by EUCAST and can be accessed freely on the EUCAST website at: www.eucast.org. EUCAST recommendations are frequently updated and the latest versions are available at: www.eucast.org.

In a recent submission to EUCAST, the same modelling approach and clinical data, as used by Roepcke *et al.*,^79^ was applied to estimate the probability of attaining PK/PD targets across a range of MIC values for *C. auris* (0.004 to 16 mg/L) determined using the modified EUCAST E.Def 7.4 broth microdilution methodology containing 0.002% Tween 20.^68,80^ In these simulations, after a single 400 mg rezafungin loading dose, the non-clinical PK/PD *f*AUC_0–168 h_/MIC target associated with net fungal stasis was 127 and the *f*AUC_0–168 h_/MIC target for a 1-log_10_ drop in colony-forming units from baseline was 307 (*Figure 5*). For *C. auris*, the simulations indicated that probability of target attainment following a single 400 mg dose of rezafungin was ≥95% for net fungal stasis up to EUCAST-defined MIC values of ≤0.06 mg/L. The differences in these results compared with those reported by Roepcke *et al.*^79^ are due to the different methodologies used (CLSI versus modified EUCAST E.Def 7.4 with 0.002% Tween 20).^79^

## Conclusions

The increasing prevalence of *C. auris* infections, which are often resistant to multiple classes of antifungal drugs and associated with high rates of mortality, poses a substantial worldwide health concern. Encouragingly, the potent activity of rezafungin against *C. auris* isolates and infections has been demonstrated in multiple *in vitro* and *in vivo* non-clinical experiments, and in PK/PD target attainment estimations utilizing clinical data. The *in vitro* and *in vivo* antifungal activity of rezafungin against *C. auris* isolates and infections appears to be as good as, or better than, that seen with other licensed echinocandins, and similar to that observed in other *Candida* species. Moreover, with a stable molecular structure and prolonged half-life that enables infrequent front-loaded dosing, rezafungin can achieve higher initial plasma concentrations than other echinocandins; this can result in faster mycological clearance and has the potential to reduce the risk of antifungal resistance development. As with other echinocandins, the *in vitro* activity of rezafungin appears to be diminished in *fks*-mutant isolates.

Although evidence suggests that rezafungin is likely to benefit patients with *C. auris* infections, this is based on non-clinical or modelling studies. Clinical data are required to validate the efficacy of rezafungin in the treatment of candidemia and IC infections caused by *C. auris*. Even in the absence of clinical data, the CLSI recently approved a proposal on 20 January 2024 for a susceptible MIC breakpoint of 0.5 mg/L for rezafungin against *C. auris*.^30,81^ The population PK modelling data for rezafungin that utilized CLSI methodology (described above and illustrated in *Figure 4*^79^) estimated an MIC epidemiological cut-off value of 1.0 mg/L for *C. auris* (based on the MIC distribution) and ≥90% probability of non-clinical PK/PD target attainment for net fungal stasis and 1-log_10_ cfu reductions at MIC values of ≤1 and ≤0.25 mg/L, respectively, after a single 400 mg rezafungin loading dose. Although these findings were integral to the CLSI submission, the rationale for the CLSI-susceptible breakpoint of 0.5 mg/L for rezafungin against *C. auris* was based on evaluation of a broader data set incorporating empirically determined rezafungin MIC distributions for *C. auris* and other *Candida* species isolates, data from *in vitro* and *in vivo* PK/PD models and clinical trial data (from healthy subjects and patients with non-*auris Candida* infections).^30,81^

A sub-analysis of the Phase 3 ReSTORE study showed rezafungin to be clinically active against candidemia/IC infections regardless of baseline *Candida* species, in terms of global cure and mycological eradication by Day 14 and 30-day all-cause mortality, although no infections were caused by *C. auris*.^30^ Furthermore, outcomes across *Candida* species did not appear to be affected by rezafungin MIC values. As the non-clinical data reviewed here suggest that rezafungin exhibits similar potent anti-*C. auris* activity to other *Candida* species, it would be reasonable to anticipate comparable clinical efficacy against *C. auris* candidemia/IC infections. Thus, along with other new antifungals, such as fosmanogepix and ibrexafungerp, rezafungin could prove to be an effective option for managing difficult-to-treat cases of candidemia and IC caused by *C. auris*. Given its reduced activity against echinocandin-resistant *fks*-mutant isolates, rezafungin is most likely to be effective against infections caused by wild-type isolates and strains that are resistant to azoles and/or AMB. Future prospective clinical studies of rezafungin in patients with IC/candidemia due to *C. auris* are warranted to define its efficacy (including efficacy against infections caused by *fks*-mutant isolates) and establish *C. auris*-specific susceptibility breakpoints.

## Transparency declarations

J.B.L., S.F. and V.O. are employees and shareholders of Cidara Therapeutics, Inc. D.A. reports no conflicts of interest. M.R. and J.A.A. are employees of Melinta Therapeutics, LLC. P.G.P. received research grants from Melinta, Cidara, F2G, Gilead and Scynexis; and was a scientific consultant for F2G, Melinta, ISHA Pharma, GSK, Basilea and Scynexis. M.C. is an employee of Element Iowa City (JMI Laboratories), who was contracted to perform services in 2023–24 for AbbVie, Inc., Adamed Pharma S.A., AimMax Therapeutics, Allecra Therapeutics, Amicrobe, Inc., AN2 Therapeutics, Inc., Apnimed, Astellas Pharma, Inc., Basilea Pharmaceutica AG, Baxis Pharmaceuticals, Inc., Beckman Counter, Inc., bioMérieux, Biosergen AB, Blacksmith Medicines, Bugworks, Carnegie Mellon University, Center for Discovery and Innovation, Cerba Research NV, Cidara Therapeutics, Cipla USA Inc., ContraFect Corporation, CorMedix Inc., Crestone, Inc., Discoveric Bio Beta Ltd, Dr Falk Pharma Gmbh, Entasis Therapeutics, Evopoint Biosciences, Fedora Pharmaceuticals, Forge Therapeutics, GARDP Foundation, Genentech, Gilead Sciences, Inc., GlaxoSmithKline plc, Harvard University, Institute for Clinical Pharmacodynamics, Iterum Therapeutics plc, Janssen Biopharma, Johnson & Johnson, Kbio, Inc., Lakewood-Amedex Inc., Locus Biosciences, Inc., McGill University, Medpace, Inc., Meiji Seika Pharma, Melinta Therapeutics, Menarini Group, Merck & Co., MetCura Pharmaceuticals, Inc., MicuRx Pharmaceutical Inc., Mundipharma International Ltd, Nabriva Therapeutics, National Cancer Institute, National Institutes of Health, NovoBiotic Pharmaceuticals, LLC, Ohio State University, Omnix Medical Ltd, Paratek Pharmaceuticals, Pfizer, Inc., PPD Global Central Labs, LLC, Pulmocide Ltd, Qpex Biopharma, Inc., Revagenix, Inc., Roche Holding AG, Scynexis, Inc., Seed Health, SeLux Diagnostics, Shionogi & Co., Ltd, Spero Therapeutics, Sumitovant Biopharma, Inc., Swedish National Reference Laboratory, TenNor Therapeutics, ThermoFisher Scientific, US Food and Drug Administration, University of Wisconsin, VenatoRx Pharmaceuticals, Wockhardt, and Zoetis, Inc. G.R.T. reports research support from, and consultancy for, Astellas, Basilea, Cidara, F2G, Mundipharma, Melinta and Pfizer.
